# Local Analysis of Heterogeneous Intracellular Transport: Slow and Fast Moving Endosomes

**DOI:** 10.3390/e23080958

**Published:** 2021-07-27

**Authors:** Nickolay Korabel, Daniel Han, Alessandro Taloni, Gianni Pagnini, Sergei Fedotov, Viki Allan, Thomas Andrew Waigh

**Affiliations:** 1 Department of Mathematics, The University of Manchester, Manchester M13 9PL, UK; daniel.han@manchester.ac.uk (D.H.); sergei.fedotov@manchester.ac.uk (S.F.); 2 School of Biological Sciences, The University of Manchester, Manchester M13 9PT, UK; viki.allan@manchester.ac.uk; 3 Biological Physics, Department of Physics and Astronomy, The University of Manchester, Manchester M13 9PL, UK; 4 CNR—Consiglio Nazionale delle Ricerche, Istituto dei Sistemi Complessi, Via dei Taurini 19, 00185 Roma, Italy; alessandro.taloni@isc.cnr.it; 5 BCAM—Basque Center for Applied Mathematics, Mazarredo 14, 48009 Bilbao, Spain; gpagnini@bcamath.org; 6 Ikerbasque—Basque Foundation for Science, Plaza Euskadi 5, 48009 Bilbao, Spain

**Keywords:** heterogeneous, anomalous diffusion, endosomes

## Abstract

Trajectories of endosomes inside living eukaryotic cells are highly heterogeneous in space and time and diffuse anomalously due to a combination of viscoelasticity, caging, aggregation and active transport. Some of the trajectories display switching between persistent and anti-persistent motion, while others jiggle around in one position for the whole measurement time. By splitting the ensemble of endosome trajectories into slow moving subdiffusive and fast moving superdiffusive endosomes, we analyzed them separately. The mean squared displacements and velocity auto-correlation functions confirm the effectiveness of the splitting methods. Applying the local analysis, we show that both ensembles are characterized by a spectrum of local anomalous exponents and local generalized diffusion coefficients. Slow and fast endosomes have exponential distributions of local anomalous exponents and power law distributions of generalized diffusion coefficients. This suggests that heterogeneous fractional Brownian motion is an appropriate model for both fast and slow moving endosomes. This article is part of a Special Issue entitled: “Recent Advances In Single-Particle Tracking: Experiment and Analysis” edited by Janusz Szwabiński and Aleksander Weron.

## 1. Introduction

Intracellular transport of organelles, such as endosomes, has been described by anomalous diffusion caused by different mechanisms [1,2]. Various models have been proposed to describe it, such as fractional Brownian motion (FBM), continuous time random walks and fractional Langevin equations [3]. However, which of these models is the best is a current topic of much debate.

To decipher which mechanism is at work and determine the appropriate mathematical model to describe it, a large ensemble of trajectories is necessary. Modern experimental techniques facilitate the tracking of large ensembles of intracellular objects for considerable amounts of time. Therefore, the extraction of meaningful statistical information from trajectories is becoming an important issue. The traditional statistical analysis of trajectories includes quantification of ensemble evolution in time and space using the ensemble-averaged mean squared displacements (EMSD), time-averaged MSD (TMSD), probability density functions of displacements and correlation functions. As the accessible measurement time in experiments increases with better live-cell microscopy techniques, the accurate analysis of single trajectories has become possible [4]. New methods of trajectory analysis were developed, such as local time-averaged MSD [5], first passage probability analysis [6,7,8] and time-averaged diffusion coefficients [9].

Improved microscopy imaging, tracking and analysis methods revealed the intrinsic spatial and temporal heterogeneity within individual trajectories of numerous biological processes [5,10,11,12,13,14,15,16,17,18]. Significant progress has also been made in analysis and interpretation of superresolution single particle trajectories [19,20,21,22,23]. Recently, individual trajectories of quantum dots in the cytoplasm of living cultured cells were found to perform subdiffusive motion of the FBM type with switching between two distinct mobility states [24]. In contrast to homogeneous systems, heterogeneous trajectories are most prominently described by broad distributions of diffusivities and anomalous exponents, an exponential probability distribution of diffusivities and a Laplace probability distribution of displacements [25]. These observations led to the development of various heterogeneous diffusion models [26,27,28,29,30,31,32,33,34,35,36,37,38,39].

Recently, the intracellular transport of endosomes in eukaryotic cells was shown to be described by spatiotemporal heterogeneous fractional Brownian motion (hFBM) with non-constant Hurst exponents [40]. By analyzing the local motion of endosomes, we found that it is characterized by power-law probability distributions of displacements and displacement increments, exponential probability distributions of local anomalous exponents and power-law probability distributions of local generalized diffusion coefficients. In this paper, we split the ensemble of endosomes into slow and fast moving vesicles, which is the main difference between this study and that of [40]. This splitting allows us to study sub-ensembles separately in addition to studying the ATP driven active transport of endosomes. In particular, there is the central question: What is the appropriate mathematical model to describe the subdiffusive transport of slow moving endosomes? By analyzing locally the slow and fast endosomal trajectories, we find that both are characterized by exponential distributions of anomalous exponents and power-law distributions of generalized diffusion coefficients. This suggests that hFBM is an appropriate model for both slow and fast endosomes.

Endosome trajectories are composed of segments of active and passive motion, and therefore they could be further decomposed into directed runs and random motion. We segmented endosomal trajectories in this way in [10]. In this study, we separated endosome trajectories into superdiffusive trajectories and subdiffusive trajectories for their whole duration. Subdiffusive trajectories do not contain segments of directed movement and cannot be segmented further into active and passive motion. In contrast, fast superdiffusive trajectories can be further segmented. We leave the segmentation of fast trajectories into directed runs and random motion for future work.

## 2. Materials and Methods

### 2.1. Experimental Trajectories

**Figure A1:**
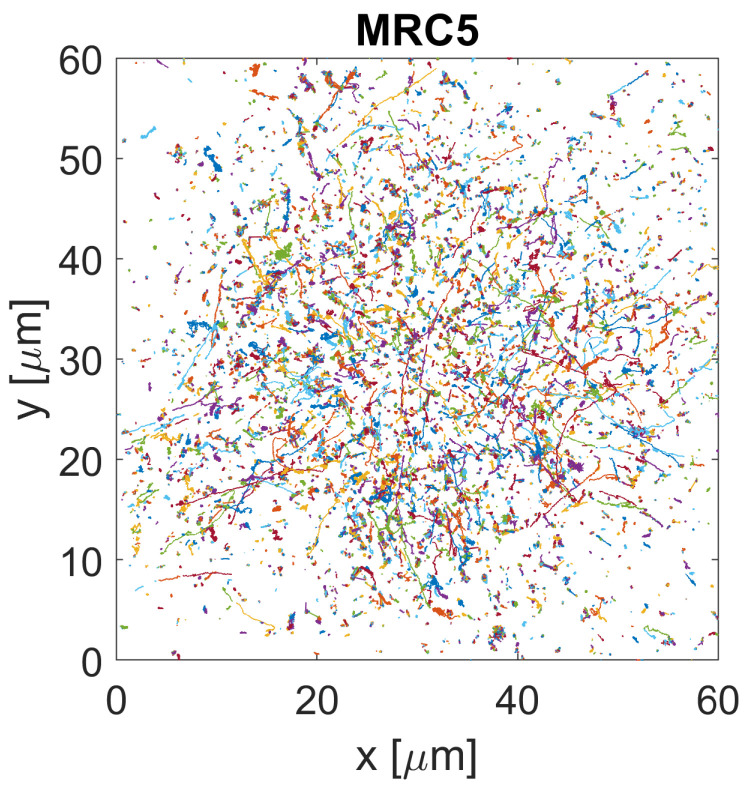
An example of the experimental endosomal trajectories (30,000 trajectories are shown).

We studied a large ensemble of two dimensional experimental trajectories, r(t)={x(t),y(t)}, of early endosomes in a stable MRC5 cell line expressing GFP-Rab5. Trajectories were obtained from tracking wide-field fluorescence microscopy videos (see [10] for experimental details). We studied 103,361 experimental trajectories of early endosomes, the same data acquired in [10]. Three live-cell microscopy videos of MRC5 cells stably expressing GFP-Rab5 could be found in the Supplementary Material (https://zenodo.org/record/5106450#.YPBsEuhKhPY, accessed on 23 July 2021). An example of experimental trajectories is shown in Figure A1. The endosomes were tracked using an automated tracking software (AITracker, based on a convolutional neural network) [41]. Currently, it is not yet feasible to determine the diameter of endosomes in these experiments, because they are diffraction limited. Thus, it was possible to track the centers of endosomes with sub-pixel accuracy, but not the sizes of the smaller endosomes (less than 200 nm). The duration of all trajectories, *T*, has a good fit to a power law distribution, $T^{-1.85}$ [40], which is a manifestation of the heterogeneity of the trajectories. Slow moving endosomes stay longer within the observation volume and therefore have longer trajectories than fast moving endosomes, leading to the emergence of the power-law probability distribution for the trajectories’ duration.

### 2.2. Splitting of Ensemble into Slow and Fast Moving Endosomes

We split ensemble of trajectories into slow and fast moving endosomes using the distance traveled by endosomes:

(1) $$R\left(t\right)=\sqrt{{\left(x\left(t\right)-x\left(0\right)\right)}^{2}+{\left(y\left(t\right)-y\left(0\right)\right)}^{2}}.$$

**Figure 1:**
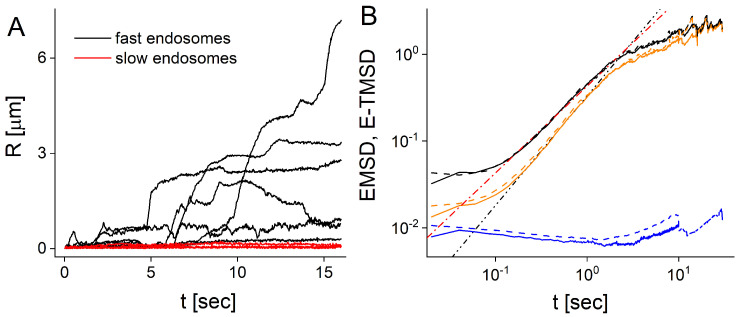
Endosomes are split into slow and fast moving: (**A**) Distance R(t) traveled by fast (black curves) and slow (red curves) endosomes (nine sample experimental trajectories are shown). Most experimental trajectories possess active motion visible as a rapid increase or decrease of *R*; (**B**) EMSDs (solid curves) and E-TMSDs (dashed curves) of fast (black curves) and slow (blue curves) endosomes compared with EMSD and E-TMSD of all trajectories (orange curves). The dashed-dotted and dashed-double-dotted lines represent $t^{1.26}$ and *t* functions.

Trajectories which possess active motion have periods of rapid increase or decrease of *R* (Figure 1A). Fast trajectories which have active motion are defined as max{R(t)}>ϵ and slow trajectories which exhibit only passive motion are defined by max{R(t)}<ϵ. Here, max{R(t)} denotes the maximum values of R(t) attained in the time interval (0,t) and ϵ is the threshold. We choose the threshold ϵ=0.25μm. In the Appendix, we show that changing the threshold to ϵ=0.2μm in the splitting does not qualitatively change the results. Therefore, we define fast moving endosomes as those that, in the time interval (0,t), experienced at least one period of active motion and the maximum distance travelled from the origin exceeds the threshold of ϵ=0.25μm. Otherwise, an endosome is defined as slow moving. Small variations of the threshold value do not affect the EMSDs of slow and fast moving endosomes, which suggests that the splitting method is robust (Figure A3).

**Figure A2:**
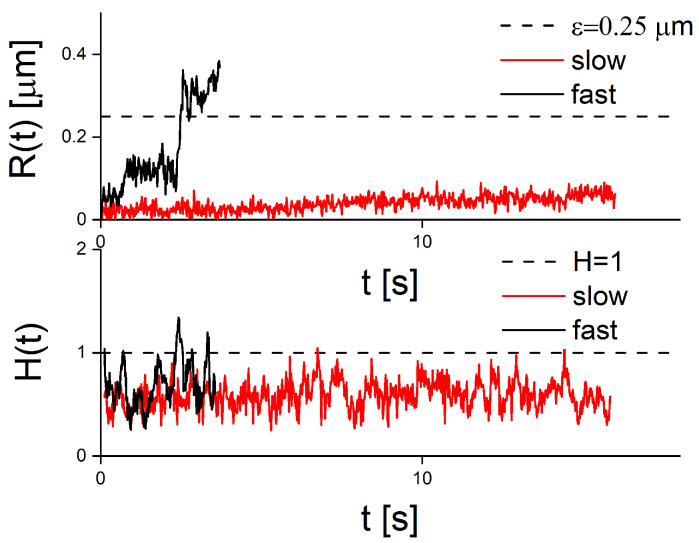
An example of experimental trajectories of slow and fast moving endosomes obtained using the maximum distance traveled R(t) (**top**) and the time-dependent anomalous exponent H(t) estimated with the neural network (**bottom**).

**Figure A3:**
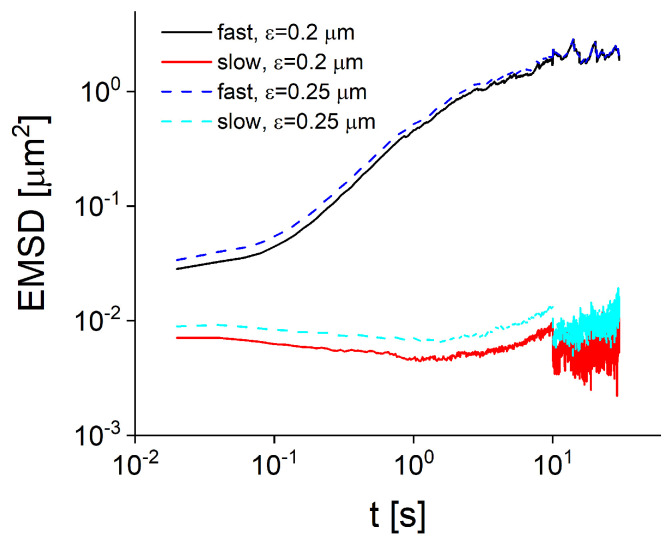
The EMSD of experimental trajectories of slow and fast moving endosomes calculated with the splitting method, which uses the maximum distance traveled R(t) for two values of the threshold: ϵ=0.2 μm and ϵ=0.25 μm.

**Figure A4:**
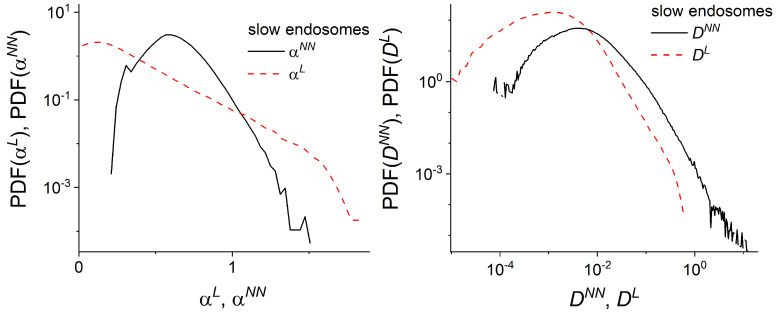
Slow endosomes. (**Left**) Distribution of anomalous exponents $\alpha^{NN}$ of slow moving endosome trajectories (the solid curve) compared with the distribution of local anomalous exponents $\alpha^{L}$ of slow moving endosome trajectories (the dashed curve); (**Right**) Distribution of generalized diffusion coefficients $D^{NN}$ of slow moving endosome trajectories (the solid curve) compared with the distribution of local generalized diffusion coefficients $D^{L}$ of slow moving endosome trajectories (the dashed curve).

Changing the splitting threshold from max{R(t)}=0.25μm to max{R(t)}=0.2μm, the increase of the number of slow trajectories was 12%. Therefore, in addition to the method of splitting trajectories which uses the minimum travelled distance, we also tested a second method, which makes use of the time-dependent Hurst exponent H(t) neural network (NN) estimate at the single trajectory level [10]. The procedure is as follows: (1) estimate the time-dependent anomalous exponent $\alpha^{NN}$ using the NN; (2) if the anomalous exponent $\alpha^{NN}$ is superdiffusive $\alpha^{NN}\left(t\right)>1$ for more than 4 consecutive time points, the endosome is considered as fast moving. Otherwise, the endosome is labeled as slow moving (see Figure A2). The correct implementation of the NN procedure requires a minimum time window [10] that is larger than the duration of some of the endosomal trajectories. Hence, short trajectories were discarded in this analysis. The similarity of the distributions of generalized diffusion coefficients (Figure A3B and Figure A4B) suggests that the chosen threshold max{R(t)}=0.25μm was reasonable. Alternative methods of binary classification could be performed using the first passage probability analysis [7] or implementing the normalized radius of gyration of each trajectory [42].

### 2.3. Ensemble and Time Averaged Mean Squared Displacements

From the two dimensional experimental trajectories r(t)={x(t),y(t)}, we calculated the ensemble-averaged mean squared displacement (EMSD) as

(2) $$\mathrm{EMSD}\left(t\right)=\frac{{\langle r\rangle }^{2}\left(t\right)}{l^{2}},$$

where *l* is the length scale which we choose l=1 μm,

(3) $${\langle r\rangle }^{2}\left(t\right)=\langle {\left(x_{i}\left(t\right)-x_{i}\left(0\right)\right)}^{2}+{\left(y_{i}\left(t\right)-y_{i}\left(0\right)\right)}^{2}\rangle ,$$

where the angular brackets denotes averaging over an ensemble of trajectories, $\langle A\rangle =\sum_{i=1}^{N}A_{i}/N$ and *N* is the number of trajectories in the ensemble.

By fitting the EMSD to power law functions, the anomalous exponent α and the generalized diffusion coefficient $D_{\alpha }$ can be extracted using

(4) $$\mathrm{EMSD}\left(t\right)=4D_{\alpha }{\left(\frac{t}{\tau }\right)}^{\alpha },$$

where α and $D_{\alpha }$ are constants which characterize averaged transport properties of ensemble of endosomal trajectories. The time scale τ=1 s and the length scale l=1 μm are introduced in order to make the generalized diffusion coefficient $D_{\alpha }$ dimensionless.

The time-averaged mean squared displacement (TMSD) of an individual trajectory $\left\{x_{i},y_{i}\right\}$ of a duration *T* is calculated as:

(5) $${\mathrm{TMSD}}_{i}\left(t\right)=\frac{\bar{\delta^{2}\left(t\right)}}{l^{2}},$$

where *l* is the length scale, for which we chose l=1 μm, and

(6) $$\bar{\delta^{2}\left(t\right)}=\frac{\int_{0}^{T-t}\left(x_{i}\left(t^{\prime }+t\right)-x_{i}\left(t^{\prime }\right){)}^{2}+{\left(y_{i}\left(t^{\prime }+t\right)-y_{i}\left(t^{\prime }\right)\right)}^{2}\right)dt^{\prime }}{T-t}.$$

TMSDs of individual trajectories are averaged further over the ensemble of trajectories to get the ensemble-time-averaged MSD (E-TMSD):

(7) $$E-\mathrm{TMSD}\left(t\right)=\langle {\mathrm{TMSD}}_{i}\left(t\right)\rangle ,$$

where the angular brackets denotes averaging over an ensemble of trajectories as before.

### 2.4. Local Analysis of Endosomal Trajectories

The time-local statistical analysis was implemented as follows. We considered only the portion of a single endosomal trajectory within a window of size *W* and centered around the time *t*, i.e., (t−W/2,t+W/2). We calculated the TMSD within this chunk of trajectory only: this is the reason for the acronym L-TMSD, i.e., the local TMSD. The experimental detection of the endosomal motion is achieved with the frame rate 1/Δt s${}^{-1}$, hence t=iΔt (here, i=0,1,2,⋯ is the time index) and W=NΔt, with N>10. The first 10 points of the L-TMSD were fitted with the power-law function

(8) $$L-\mathrm{TMSD}=4D^{L}\left(t\right){\left(\frac{t^{\prime }}{\tau }\right)}^{\alpha^{L}\left(t\right)},$$

where $t^{\prime }=10\Delta t$. $\alpha^{L}\left(t\right)$ and $D_{\alpha^{L}}\left(t\right)$ are the local anomalous exponent and generalized diffusion coefficient, respectively. We iterate this procedure by shifting the time window of a single Δt (i→i+1) until the end of the experimental endosomal trace, thus obtaining $\alpha^{L}\left(t\right)$ and $D_{\alpha^{L}}\left(t\right)$ along the entire trajectory. Notice that $\alpha^{L}$ and $D^{L}$ are not constants in time and they vary, being local properties of each endosomal trajectory.

### 2.5. The Time and Ensemble-Time Averaged Velocity Auto-Correlation Functions

The time averaged auto-correlation function (TVACF) along a single trajectory is defined as:

(9) $${\mathrm{TVACF}}_{i}\left(t\right)=\frac{\int_{0}^{T-t-\tau }\vec{v}\left(t^{\prime }+t\right)\vec{v}\left(t^{\prime }\right)dt^{\prime }}{T-t-\tau },$$

where $\vec{v}=\frac{\vec{r}\left(t+\tau \right)-\vec{r}\left(t\right)}{\tau }$. TVACFs of individual trajectories are averaged further over the ensemble of trajectories to get the ensemble-time averaged VACF (E-TVACF):

(10) $$E-\mathrm{TVACF}\left(t\right)=\langle {\mathrm{TVACF}}_{i}\left(t\right)\rangle ,$$

where the angular brackets denotes averaging over an ensemble of trajectories. The velocity autocorrelation function was suggested as a tool to distinguish between subdiffusion models [43].

## 3. Results

We split the ensemble of endosomes into slow and fast moving vesicles using the two methods described above (see Methods, Figure 1A and Figure A2). For both slow and fast endosomes, the EMSDs and E-TMSDs show similar behavior, which suggests ergodicity (see Methods and Figure 1B). MSDs of slow endosomes are not increasing in time, which confirms that these trajectories have no active periods of motion. Surprisingly, we found that both EMSDs and E-TMSDs of slow endosomes are decreasing functions of time, which to our knowledge has never been observed before. We explain this behavior in terms of the coupling between the average diffusivities of slow trajectories and their duration (see Figure 4 and the discussion below). Conversely, MSDs of fast endosomes are increasing functions of time in the intermediate time scale (0.2,2) s. The anomalous exponent extracted from EMSD or E-TMSD of fast endosomes is α≃1, smaller than the anomalous exponent obtained by considering all trajectories without distinction into fast or slow, i.e., α≃1.26. Notice that two subdiffusive regimes characterize the MSD time behavior for fast and all trajectories. The first, at small time scales ($t\leq 10^{-1}$ s), can be attributed to the measurement errors [44,45,46]. The second, at longer time scales (t>10 s), was shown to be spurious and originate from the coupling of the trajectories’ duration and their diffusivities [40,47]. We suggest that, due to this coupling, the anomalous exponents deduced from the power-law fit of EMSD and E-TMSD, do not capture the essential characteristics of the endosome superdiffusive motility, nor shed light on its fundamental aspects. Therefore, to reveal the effect of the duration of trajectories on the statistical analysis, we consider only trajectories longer than a certain threshold *T* [40].

**Figure 2:**
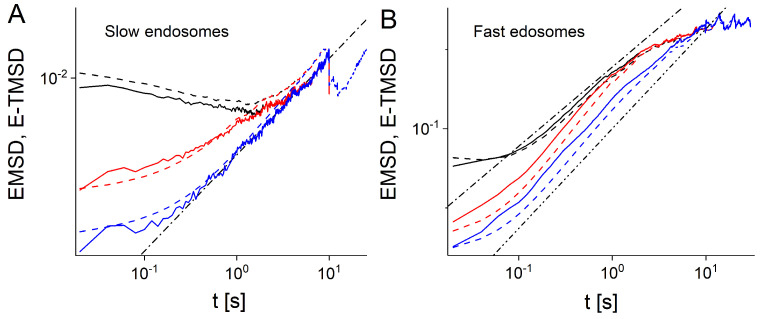
EMSDs and E-TMSDs (solid and dashed curves) of experimental trajectories of slow (**A**) and fast moving endosomes (**B**). Black curves correspond to T→∞ s. Red and blue curves represent EMSDs and E-TMSDs of experimental trajectories which have duration longer than 2 and 8 s, respectively. The dashed-dotted line in (**A**) represents the function $t^{0.5}$. In (**B**), the dashed-dotted line and the dashed-double-dotted line represent the linear, *t*, and super-linear, $t^{1.26}$, functions, respectively.

Figure 2A,B shows the EMSDs and E-TMSDs of slow and fast endosomes, considering only experimental trajectories with duration longer than *T* seconds (2 or 8 s). Unlike the slow moving endosomes, the MSDs of fast vesicles (Figure 2B) present similar qualitative behaviors by choosing T=2 s, T=8 s or no *T* at all (all the fast molecules considered as in Figure 1B, black curve). However, in the intermediate regime, the superdiffusive behavior becomes more and more apparent, $\propto t^{1.26}$, and stable. In [40] we found that this process is described by the space-time heterogeneous FBM with the Hurst exponent *H* that randomly switches between persistent H>0.5 and anti-persistent regimes H<0.5, together with the coupling between the diffusivity and duration of trajectories which account for spurious subdiffusion at longer time scales. Moreover, the EMSD curves obtained for T=2 s and T=8 s deviates considerably from the corresponding E-TMSD curves.

The MSDs of slow endosomes (Figure 2A) display very different, but ergodic, behavior. For 0.01<t<2 s, the MSDs of all slow endosomes decreases in time. On the other side, the MSDs of the sub-ensembles of slow endosomes with T=2 s and T=8 s reveal subdiffusive trends with α∼0.5. As in the case of fast moving endosomes, we argue that this behavior is due to the coupling between the diffusivity and duration of trajectories. Therefore, we attempt to confirm this hypothesis, by performing simulations of an ensemble of heterogeneous FBM trajectories with constant Hurst exponent H=0.25 (see Figure 4).

**Figure 3:**
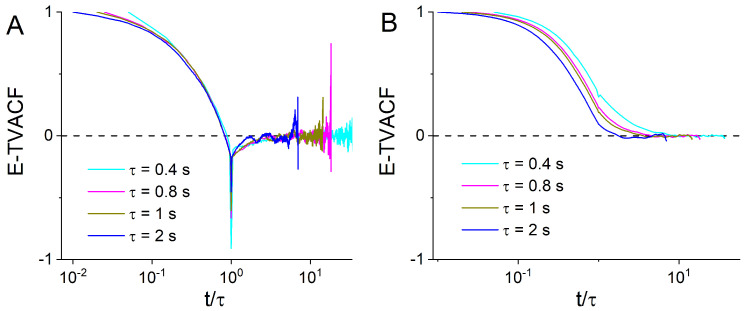
Time-ensemble averaged VACF (E-TVACF) of experimental trajectories of slow (**A**) and fast endosomes (**B**) calculated for different τ given in the legend.

The velocity auto-correlation functions (VACF) also confirm the effectiveness of this simple threshold splitting (Figure 3A,B). Indeed, slow and fast endosomes have very different VACFs. Ensemble-time averaged VACFs (E-TVACFs) of fast endosomes (Figure 3B) are positive as expected for superdiffusive motion. In contrast, E-TVACFs of slow endosomes have negative dips at t=τ and approach zero from negative values (Figure 3A). Such behavior is characteristic of FBM and the generalized Langevin equation but cannot be reproduced by the CTRW model [3].

**Figure 4:**
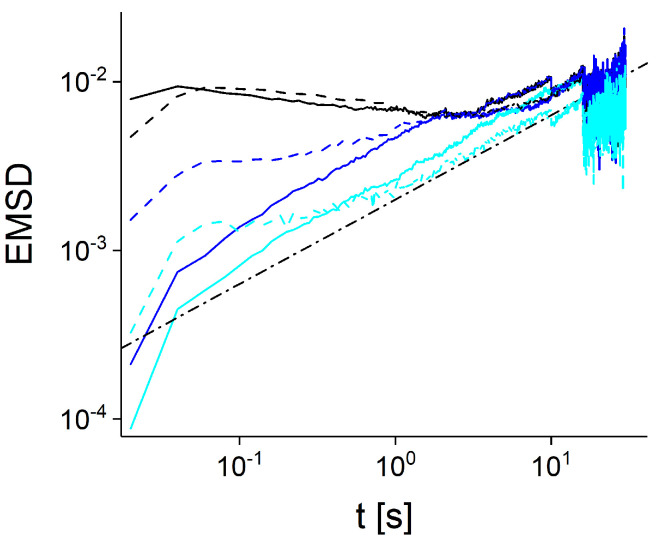
EMSDs calculated for simulated hFBM trajectories (solid lines) as a function of time interval. Black curves correspond to EMSDs of all trajectories of slow endosomes and hFBM, blue curves are EMSDs of trajectories longer than T=2 s and cyan curves are EMSDs of trajectories longer than T=8 s. The subdiffusive behavior with the anomalous exponent α=0.5 is shown as the dashed-dotted line. The EMSDs of slow experimental endosomal trajectories are shown for comparison (dashed lines). Notice that hFBM trajectories were simulated without external noise (measurement error), which led to discrepancy between simulated and experimental EMSDs at small time scale.

To verify that heterogeneous FBM describes slow moving endosomes, we simulated an ensemble of hFBM trajectories. Individual hFBM trajectories were simulated with constant Hurst exponent H=0.25. For standard FBM, this would correspond to subdiffusive MSDs, $\langle r^{2}\left(t\right)\rangle$∼$t^{2H}$$∼t^{0.5}$. The duration of hFBM trajectories was drawn from the power-law distribution ϕ(T)∼$T^{-1.85}$, in accordance with the experimental evidence [40]. The generalized diffusion coefficients were chosen inversely proportional to the duration of trajectories, i.e., *D*∼$T^{-0.6}$. As shown in Figure 4, the EMSDs of hFBM trajectories agree well with the experimental data.

**Figure A6:**
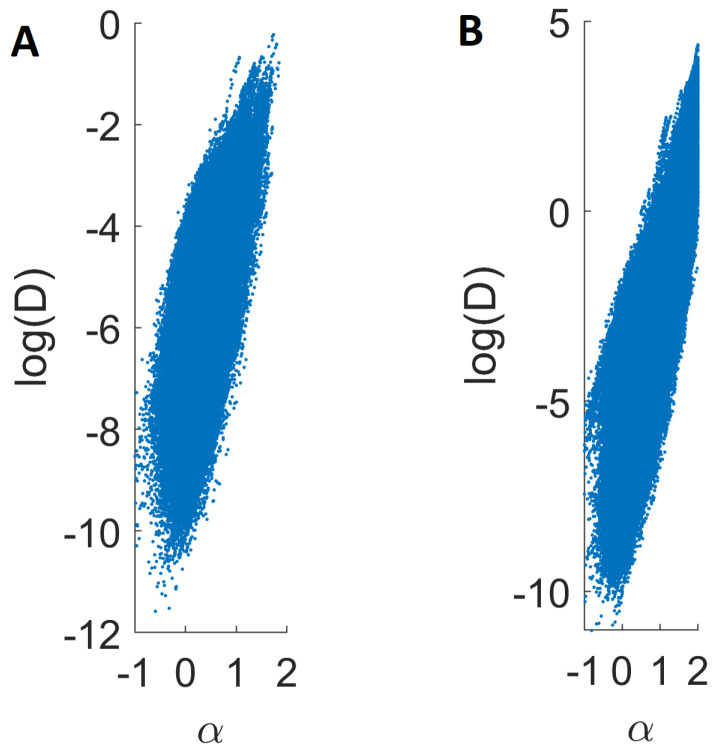
Correlation between local anomalous exponents $\alpha^{L}$ and local generalized diffusion coefficients $D^{L}$ for slow (**A**) and fast (**B**) moving endosomes.

We next implemented the local analysis [40] to better characterize the slow and fast endosomal dynamics. We calculated the local TMSDs (L-TMSD) for each experimental trajectory at various times *t* ( Methods). From the fit of L-TMSD to Equation (8), we extracted the local anomalous exponents $\alpha^{L}\left(t\right)$ and the local generalized diffusion coefficients $D_{\alpha^{L}}\left(t\right)$ for slow and fast endosomes separately. The local anomalous exponents $\alpha^{L}\left(t\right)$ and the local generalized diffusion coefficients $D_{\alpha^{L}}\left(t\right)$ appear to be positively correlated both for slow and fast endosomes (see Figure A6). The origin of these correlations is not known and will be investigated in future publications. In [40], we found that PDFs of local anomalous exponents and local generalized diffusion coefficients do not depend on the window size or the time *t* (stationary) and are best fitted with exponential and power law functions, respectively.

**Figure 5:**
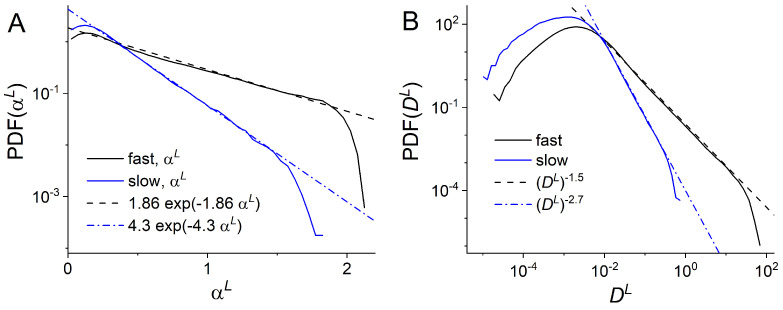
Distribution of local anomalous exponents $\alpha^{L}$ (**A**) and local generalized diffusion coefficients $D^{L}$ (**B**) obtained from experimental trajectories of slow and fast endosomes. The dashed and dashed-dotted lines are best fits to exponential (**A**) and power-law PDFs (**B**). In (**A**), they correspond to $1.86\exp(-1.86\alpha^{L})$ for PDF of $\alpha^{L}$ of fast endosomes (dashed line) and $4.3\exp(-4.3\alpha^{L})$ for PDF of $\alpha^{L}$ of slow endosomes (dashed-dotted line). In (**B**) they correspond to ${\left(D^{L}\right)}^{-1.5}$ for PDF of $D^{L}$ of fast endosomes (dashed line) and ${\left(D^{L}\right)}^{-2.7}$ for PDF of $D^{L}$ of slow endosomes (dashed-dotted line).

The PDFs of $\alpha^{L}$ and $D_{\alpha^{L}}$ for slow and fast endosomes are shown in Figure 5A,B. In both cases, the PDFs of $\alpha^{L}$ follow an exponential distribution, while those of $D_{\alpha^{L}}$ are best fitted with a power-law. However, the parameters characterizing the distribution shapes are very different. Furthermore the parameters for the fast endosomes’ PDFs coincide with those found by considering all experimental trajectories [40]. This is in agreement with a heterogeneous FBM model of endosomal transport [40], which describes the endosome motion as FBM with non-constant Hurst exponents.

**Figure 6:**
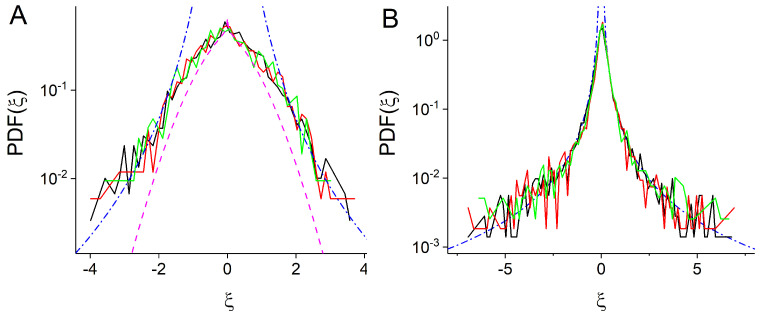
Distribution of scaled x-component of coordinate $\xi =x/\sigma_{x}$ obtained from experimental trajectories of slow (**A**) and fast (**B**) endosomes. The dashed-dotted lines correspond to power-law fit ${\left|\xi \right|}^{-1-2\gamma_{S}}$ for slow endosomes ($\gamma_{S}≃1.7$) and ${\left|\xi \right|}^{-1-2\gamma_{F}}$ for fast endosomes ($\gamma_{F}≃0.5$). In (**A**), we also compare PDF of slow endosomes with the analytical propagator for obstructed diffusion (dashed line) [48].

Finally, we calculated propagators of experimental trajectories for slow and fast endosomes (Figure 6). Using the power-law forms of distributions of local generalized diffusion coefficients of slow $p_{S}\left(D^{L}\right)$∼${\left(D^{L}\right)}^{-1-\gamma_{S}}$ and fast $p_{F}\left(D^{L}\right)∼{\left(D^{L}\right)}^{-1-\gamma_{F}}$ endosomes with $\gamma_{S}≃1.7$ and $\gamma_{F}≃0.5$ (Figure 5), we fit the propagators with the propagators of hFBM, PDF(ξ)∼${\left|\xi \right|}^{-1-2\gamma }$ with $\gamma =\gamma_{S}$ for slow endosomes and $\gamma =\gamma_{F}$ for fast endosomes (see Supplementary Note and [33]). For slow endosomes (Figure 6A), we also compare the experimental PDFs with the analytical propagator for obstructed diffusion in two dimensions, $\xi^{-0.108}{\exp(-|\xi |}^{1.65})$ [48].

## 4. Discussion

In this paper, we extend our investigation of the heterogeneous intracellular transport of endosomes based on the local analysis of experimental trajectories [40]. Individual endosomes move for long distances in a heterogeneous way with short bursts of directed motility, interspersed with periods of subdiffusive motion [49,50]. The heterogeneous character of this motion is also manifested as some endosomes are less motile than others. Some endosomes look as if they are jiggling in one position for the whole period of observation. Therefore, we split the ensemble of trajectories into slow and fast moving endosomes. The distinct time behavior of mean squared displacements and velocity auto-correlation functions confirm the effectiveness of these methods. The splitting allowed us to study passive subdiffusive and active superdiffusive transport of endosomes separately.

Comparing the behavior of fast endosomes (MSDs, VACFs and propagators) to the behavior of the entire ensemble, we find that they are most consistent with FBM models [40]. Therefore, we conclude that fast endosomes follow heterogeneous FBM [40]. The ergodicity (Figure 2A) and the VACF (Figure 3A) suggest that slow endosomes are also described by the hFBM or heterogeneous generalized fractional Langevin equation motion. For slow endosomes, crowding and obstruction effects could also lead to subdiffusive behavior [2,4]. It is known that obstructed diffusion has many similarities with FBM such as stationarity of the increments and the equivalence of the time and ensemble MSDs [48,51]. The propagators provide a clear way to distinguish obstructed diffusion from FBM. Therefore, we calculated propagators of experimental slow endosomes and compared them with analytical prediction for the propagator of obstructed diffusion and prediction of heterogeneous fBM. The results shown in Figure 6 indicate that slow endosomes follow hFBM at longer time scales, while on smaller scales obstructed diffusion likely contributes to their subdiffusive behavior as well. Crowding effects remain as a possible source of anomalous diffusion of slow endosomes. Recently, in numerical simulations, lipids in crowded conditions of the membrane were shown to be multifractal and anomalous. The dynamics was no longer described by the mechanism consistent with the fractional Langevin equation or by any single known mechanism. Instead, the motion was found to be non-Gaussian and heterogeneous, yet maintains its ergodic properties [52], which is similar to what we observed for experimental trajectories of slow endosomes.

Both slow and fast endosomal trajectories are found to be highly heterogeneous in space and time. The spatial heterogeneity in the form of coupling between endosome diffusivity and duration of endosome trajectory explains the behavior of the MSDs. Longer trajectories have smaller generalized diffusion coefficients since in experiments slowly moving endosomes with smaller diffusion coefficients stay longer in the field of view, having longer durations. For slow and fast endosomes, we can conclude that EMSD and E-TMSD are not adequate to describe the large heterogeneity exhibited in space and time. Therefore, we applied a time local analysis of individual trajectories.

From the local analysis, we found that slow and fast endosomal trajectories are both characterized by exponentially distributed anomalous exponents and power-law distributed generalized diffusion coefficients. However, the parameters of these distributions are different. Although the factors that cause the power-law distributed generalized diffusion coefficients for slow and fast endosomes could be different, some common factors can exist. One of them could be the scale free properties of endosomal networks [53]. Hence, the differences in endosome diameters could generate distinct diffusive properties intrinsic to each endosome. Heterogeneous diffusion generated by the fluctuations of molecular size was found in single-molecule experiments within the cell [14,18,42]. Another common factor promoting power-law distributions of generalized diffusion coefficients could be non-specific interactions with the endoplasmic reticulum or other organelles and large intracellular structures. Recently, non-specific interactions were shown to generate heterogeneous diffusion of nanosized objects in mammalian cells [47].

Our analysis of endosomal transport would be valuable for both fundamental cell biology and nanomedicine applications such as drug and gene delivery. In these applications, nanoparticles are often used as cargo-carrying vesicles, which in turn utilize the endosomal network for their intracellular transport. For example, gold nanoparticles were shown to cluster inside endosomes and move via sub- and superdiffusion [54]. Our results would also be useful for the nanoparticle enhanced radiation therapy of cancer [55,56,57] where clusters of nanoparticles inside endosomes are used for dose enhancement.

In the future, we expect microscopy techniques will improve in tandem with tracking algorithms, providing datasets with larger ranges of time scales and improved resolution. Thus, further subclassification of ensembles of endosomal tracks (beyond the binary fast and slow separation) will become possible towards the ultimate goal of single molecule specificity. Increasing the dynamic range (to submillisecond time scales) will allow the stepping motion of the motor proteins (kinesin and dynein) attached to microtubules to be connected with the spectra of α and $D_{\alpha }$ for the fast moving endosomes at a fundamental level.

## Figures and Tables

**Figure A5:**
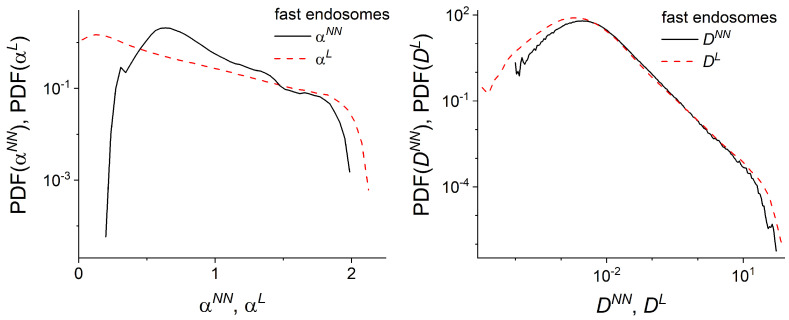
Fast endosomes. (**Left**) Distribution of anomalous exponents $\alpha^{NN}$ of fast moving endosome trajectories (the solid curve) compared with the distribution of local anomalous exponents $\alpha^{L}$ of slow moving endosome trajectories (the dashed curve); (**Right**) Distribution of generalized diffusion coefficients $D^{NN}$ of fast moving endosome trajectories (the solid curve) compared with the distribution of local generalized diffusion coefficients $D^{L}$ of fast moving endosome trajectories (the dashed curve).

## Data Availability

The data that support the findings of this study are available from the corresponding author upon reasonable request.

## Supplementary Materials

The following are available online at https://zenodo.org/record/5106450#.YPBsEuhKhPY, Video S1: Videos for MRC5 cells stably expressing GFP-Rab5.

## Appendix A

Figure A1: An example of the experimental endosome trajectories measured in MRC5 cells stably expressing GFP-Rab5. See the main text for details.

Figure A2: Two splitting methods used to separate endosome trajectories into slow and fast moving endosomes. The first method uses the maximum distance traveled R(t). The second method uses the time-dependent anomalous exponent H(t) estimated with the neural network. An example of the two trajectories is shown, which were classified as slow and fast by both methods. See the main text for details of the methods.

Figure A3: The EMSD of slow and fast moving endosomes calculated with the splitting method, which uses the maximum distance traveled R(t). Two values of the threshold ϵ produce qualitatively similar results, which suggests that the splitting method is robust against small variations of the threshold.

Figure A4: Comparison of distributions of anomalous exponents $\alpha^{NN}$ and generalized diffusion coefficients $D^{NN}$ and local anomalous exponents $\alpha^{L}$ and $D^{L}$ of slow moving endosomes. Anomalous exponents $\alpha^{NN}$ were estimated using a neural network with window size 0.26 s. The generalized diffusion coefficients $D^{NN}$ were estimated by fitting the local TMSD of the trajectory with the power law $D^{NN}t^{\alpha^{NN}}$. The distribution of $\alpha^{NN}$ has a maximum of 0.6 and decays faster than the distribution of $\alpha^{L}$. This may be because many short trajectories are missing in the NN analysis, since the NN could analyze trajectories with durations longer than its window size [10]. The distributions of generalized diffusion coefficients (right panel), on the other hand, are similar to each other.

Figure A5: Comparison of distributions of anomalous exponents $\alpha^{NN}$ and generalized diffusion coefficients $D^{NN}$ and local anomalous exponents $\alpha^{L}$ and $D^{L}$ of fast moving endosomes. Anomalous exponents $\alpha^{N}N$ were estimated using neural network with window size 0.26 s. The generalized diffusion coefficients $D^{NN}$ were estimated by fitting the local TMSD of trajectory with the power law $D^{NN}t^{\alpha^{NN}}$. The distributions of anomalous exponents (Left) are similar to each other, while the distributions of generalized diffusion coefficients (Right) are almost indistinguishable.

Figure A6: Local anomalous exponents $\alpha^{L}$ and local generalized diffusion coefficients $D^{L}$ are positively correlated for both slow and fast moving endosomes.
